# Nanofiber NiMoO_4_/g-C_3_N_4_ Composite Electrode Materials for Redox Supercapacitor Applications

**DOI:** 10.3390/nano10020392

**Published:** 2020-02-23

**Authors:** Kannadasan Thiagarajan, Thirugnanam Bavani, Prabhakarn Arunachalam, Seung Jun Lee, Jayaraman Theerthagiri, Jaganathan Madhavan, Bruno Georges Pollet, Myong Yong Choi

**Affiliations:** 1 Solar Energy Lab, Department of Chemistry, Thiruvalluvar University, Vellore 632 115, India; k.thiagupriya2016@gmail.com (K.T.); tbavani18@gmail.com (T.B.); 2 Electrochemistry Sciences Research Chair (ESRC), Chemistry Department, College of Science, King Saud University, Riyadh 11451, Saudi Arabia; parunachalam@ksu.edu.sa; 3 Department of Chemistry and Research Institute of Natural Sciences, Gyeongsang National University, Jinju 52828, Korea; venus272@gnu.ac.kr (S.J.L.); j.theerthagiri@gmail.com (J.T.); 4 Centre of Excellence for Energy Research, Sathyabama Institute of Science and Technology (Deemed to be University), Chennai 600119, India; 5 Hydrogen Energy and Sonochemistry Research Group, Department of Energy and Process Engineering, Norwegian University of Science and Technology (NTNU), NO-7491 Trondheim, Norway; bruno.g.pollet@ntnu.no

**Keywords:** NiMoO_4_, NiMoO_4_/g-C_3_N_4_ composite, redox supercapacitors, hydrothermal method

## Abstract

NiMoO_4_/g-C_3_N_4_ was fabricated by a hydrothermal method and used as an electrode material in a supercapacitor. The samples were characterized by XRD, FTIR, scanning electron microscopy (SEM) and transmission electron microscopy (TEM) to study the physical and structural properties of the as-prepared NiMoO_4_/g-C_3_N_4_ material. The electrochemical responses of pristine NiMoO_4_ and the NiMoO_4_/g-C_3_N_4_ nanocomposite material were investigated by cyclic voltammetry (CV), galvanostatic charge-discharge (GCD) and electrochemical impedance spectroscopy (EIS). From the CD studies, the NiMoO_4_/g-C_3_N_4_ nanocomposite revealed a higher maximum specific capacitance (510 Fg^−1^) in comparison to pristine NiMoO_4_ (203 Fg^−1^). In addition, the NiMoO_4_/g-C_3_N_4_ composite electrode material exhibited high stability, which maintained up to 91.8% capacity even after 2000 charge-discharge cycles. Finally, NiMoO_4_/g-C_3_N_4_ was found to exhibit an energy density value of 11.3 Whkg^−1^. These findings clearly suggested that NiMoO_4_/g-C_3_N_4_ could be a suitable electrode material for electrochemical capacitors.

## 1. Introduction

Owing to the environmental pollution caused by the emission of greenhouse gases from fossil fuels, researchers have been developing renewable energy sources for which new types of energy storage devices, such as fuel cells, batteries, conventional capacitors and electrochemical supercapacitors are required [1]. Among the numerous types of energy storage devices, supercapacitors are one of the most appropriate ESDs (energy storage devices), owing to their superior power density, fast reversible/irreversible time, long life span and low cost [2,3,4]. Supercapacitors are also known as new electrochemical capacitors or ultracapacitors. According to the storage phenomenon of the supercapacitor, they can be categorized as electrical double-layer capacitance (EDLCs) or pseudocapacitors. EDLCs store charges through the electrode and electrolyte interface by a diffusion/charge accumulation process, whereas in pseudocapacitors, the charges are stored through Faradaic processes (occurring in the active material) and electrolytes (during the redox reactions), thus displaying higher specific capacitance (SC) values than those seen for EDLCs [5].

Carbon-based composite materials, namely graphene, activated carbon, carbon aerogel, carbon nanotubes and carbon cloth, are the most used electrode materials in EDLCs [3,6], whereas transition metal oxides/hydroxides are used as electrode materials in pseudocapacitors. These metal oxides have attracted the attention of researchers owing to their significant characteristics such as high SC values, high power density and energy density, as well as rapid and reversible redox reactions at the electrode/electrolyte surfaces [7,8]. Efficient transition metal oxides and hydroxides reported so far include RuO_2_ [9], MnO_2_ [10], Co(OH)_2_ [11], NiO [12] and WO_3_ [13].

In recent years, ESD research has focused on metal molybdates, owing to their advantageous properties [14,15], one of which is the extensive research carried out around NiMoO_4_ owing to its enhanced electrochemical properties. Guo et al. [16] developed NiMoO_4_ nanowires loaded on a Ni foam electrode that delivered a superior SC of 1308 Fg^−1^ at 74.7 Ag^−1^. Likewise, Lin et al. [17] developed groove-like NiMoO_4_ hollow nanorods for high-performance supercapacitors, and the electrodes had a high SC of 1102 Fg^−1^ at a current density of 1 Ag^−1^. Senthilkumar et al. [18] synthesized nano α-NiMoO_4_ as a new electrode material for supercapacitors and calculated the SC at a current density of 1.2 Ag^−1^ as 1517 Fg^−1^. Jothi et al. [19] reported the synthesis of 1D α-NiMoO_4_ nanorods electrode materials for supercapacitors and found the maximum SC obtained from the electrochemical measurement was 730 Fg^−1^ at a scan rate of 5 mVs^−1^. Wang et al. [20] developed carbon-sheathed NiMoO_4_ nanowires loaded onto a Ni foam as an electrode material for supercapacitors, which exhibited maximal SC of 3070 Fg^−1^ at 2.5 Ag^−1^. Liu et al. [21] synthesized CoMoO_4_-NiMoO_4_·*x*H_2_O bundles by a simple coprecipitation method, which exhibited a maximum SC value of 1039 Fg^−1^ at a current density of 2.5 mAcm^−2^. In another study, Senthilkumar et al. [22] synthesized nanostructured β-NiMoO_4_-CoMoO_4_·*x*H_2_O composites through a solution combustion process, which exhibited an exceptional SC of 1472 Fg^−1^ at 5 mAcm^−2^. Ren et al. [23] synthesized NiMoO_4_@Co(OH)_2_ core-shell structured nanowire arrays by means of a hydrothermal process and an electrodeposition method route that led to a maximal SC value of 2122 Fg^−1^ at 4.5 Ag^−1^. In this present study, we also chose NiMoO_4_ as one of the electrode components. However, we coupled NiMoO_4_ with g-C_3_N_4_, one of the most widely investigated potential material for photocatalysis and other energy conversion devices [24,25,26]. Zhang et al. [27] prepared 3D RuO_2_/g‑C_3_N_4_@rGO aerogel composites for SCs that revealed a maximum SC value of 704.3 Fg^−1^ at 0.5 Ag^−1^. This study further addressed the potential applications of this strategy in developing 3D rGO aerogel composite for high-performance supercapacitors. Moreover, Zhang et al. [28] also developed Ni_2_P_2_O_7_ nanoarrays with the incorporation of g-C_3_N_4_ to yield efficient electrode materials, which retained 91% of SC efficiency after 1000 cycles.

Herein, we report for the first time the synthesis of NiMoO_4_/g-C_3_N_4_ by a hydrothermal method and its suitability as an electrode material for supercapacitors. It was found that the optimized NiMoO_4_/g-C_3_N_4_ composite coated on a carbon paper delivered a high SC value with superior cyclic stability, which could serve as a potential candidate for pseudocapacitor applications.

## 2. Experimental Section

### 2.1. Preparation of g-C_3_N_4_

The synthesis of g-C_3_N_4_ proceeded through a thermal polycondensation of urea. Typically, 6 g of urea was added in an alumina crucible (with a lid in order to avoid sublimation), followed by annealing at 500 °C for 2 h. After cooling naturally, a yellow material was obtained.

### 2.2. Preparation of NiMoO_4_/g-C_3_N_4_ by Hydrothermal Method

In double distilled (DD) water (40 mL), 3 mM of Na_2_MoO_4_·7H_2_O and 3 mM of NiCl_2_·6H_2_O (SDFCL, Mumbai, India) were dispersed, and then placed in an ultrasonic bath (42 kHz) for 30 min. A second solution was prepared by dispersing 10 mg of g-C_3_N_4_ in 20 mL DD water and was placed in an ultrasonic bath for 60 min. The two different solutions were poured into a Teflon-lined autoclave and annealed at 140 °C for 12 h. After this process, the autoclave was left to cool down. The sample was filtered and cleaned several times with DD water, followed by ethyl alcohol (at least three times). The obtained powder was dried overnight and annealed at 500 °C for 4 h. The mass ratio of g-C_3_N_4_ in the NiMoO_4_/g-C_3_N_4_ nanocomposite was 10 wt.%. The same method was followed to produce a pristine NiMoO_4_ nanomaterial without the addition of the g-C_3_N_4_ solution.

### 2.3. Preparation of Electrode Material

The electrode materials were fabricated by mixing either NiMoO_4_ or NiMoO_4_/g-C_3_N_4_(80%), polyvinylidine fluoride (Sigma Aldrich, St. Louis, MI, USA), a binder (10%) and a super P black (10%) conducting material (Timcal, Bironico, Switzerland) in the solvent 1-methyl-2-pyrrolidone until the slurry became a fine paste. The paste was then uniformly loaded on a carbon paper (surface area approximately 0.5 cm^2^) and dried at 80 °C overnight. The amount of electrode material was approximately 1 mg.

### 2.4. Electrochemical Measurements

The electrochemical properties of NiMoO_4_ and NiMoO_4_/g-C_3_N_4_ samples were assessed by cyclic voltammetry (CV)**,** galvanostatic charge-discharge (GCD), and electrochemical impedance spectroscopy (EIS). All electrochemical experiments were performed using an electrochemical system (CHI608E, USA) in a standard 3-electrode assembly using Ag/AgCl as a reference electrode, a Pt wire as a counter electrode (diameter of 0.5 mm, and length of 5 cm), and either the NiMoO_4_ or NiMoO_4_/g-C_3_N_4_ active material loaded onto carbon paper as a working electrode. A 6 M KOH alkaline solution was used as the electrolyte solution. The CV analyses were performed using an applied potential with a range from +0.1 V vs. Ag/AgCl to +0.55 V vs. Ag/AgCl, and the GCD analyses were performed in the potential range from +0.1 V vs. Ag/AgCl to +0.5 V vs. Ag/AgCl. The SC values were evaluated from the CVs, as described in [18]. The SC values of the NiMoO_4_ and NiMoO_4_/g-C_3_N_4_ electrode were evaluated based on GCD using Equation (1) [29],

(1) $$SC=\frac{I\Delta t}{m\Delta V}$$

in which *I* is the discharging current, *t* is discharged time, *m* is the amount of loaded electrode materials and Δ*V* is the applied potential difference.

## 3. Results and Discussion

### 3.1. XRD Analysis

**Figure 1:**
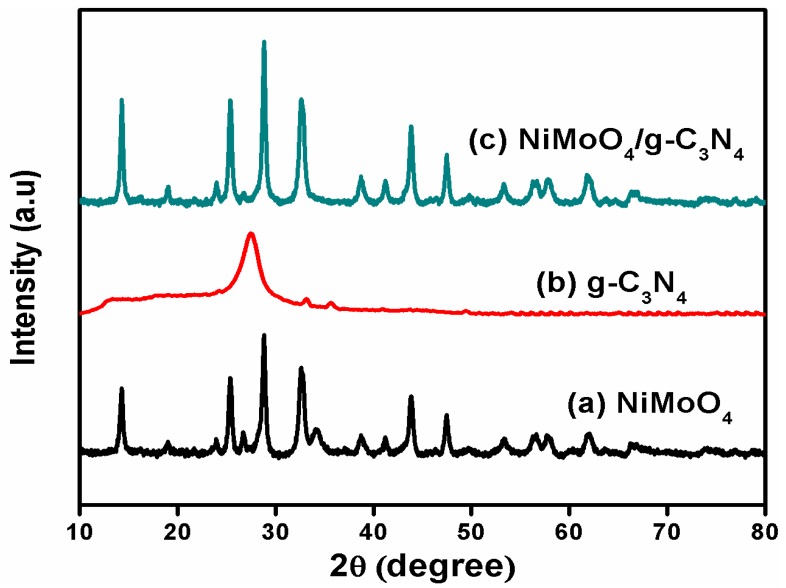
X-ray diffraction spectra of (**a**) NiMoO_4_, (**b**) g-C_3_N_4_ and (**c**) NiMoO_4_/g-C_3_N_4_.

The XRD patterns obtained for the NiMoO_4_, g-C_3_N_4_, and NiMoO_4_/g-C_3_N_4_ samples are displayed in Figure 1a–c. Figure 1a shows the diffraction lines derived from 2*θ* values of pristine NiMoO_4_ namely; 14.3°, 19.0°, 23.8°, 25.4°, 26.8°, 28.8°, 32.6°, 34.2°, 38.5°, 41.1°, 43.9°, 47.5°, 53.4°, 56.2°, 57.8°, 62.0° and 66.4°. These Bragg angles are in good agreement with JCPDS No. 45-0142, according to a monoclinic phase. The XRD of g-C_3_N_4_ exhibited two diffraction peaks at 2*θ* values 13.06° and 27.4°, which are assigned to the (100) and (002) planes of hexagonal g-C_3_N_4_ (JCPDS card No. 87-1526). Thus, the peaks which appeared at 13.06° and 27.4° are corresponding to the in-plane structural design and the interlayer stacking of the aromatic part of g-C_3_N_4_ [30]. The NiMoO_4_/g-C_3_N_4_ nanocomposite has a similar XRD pattern to that of pristine NiMoO_4,_ which can be owing to the untraceable amount of g-C_3_N_4_ present in the composite. However, it can be observed that the peak intensity of NiMoO_4_/g-C_3_N_4_ is increased by the addition of g-C_3_N_4_. Hence, the obtained XRD results support the formation of a NiMoO_4_/g-C_3_N_4_ composite.

### 3.2. Fourier Transform Infrared (FTIR) Spectroscopy

**Figure 2:**
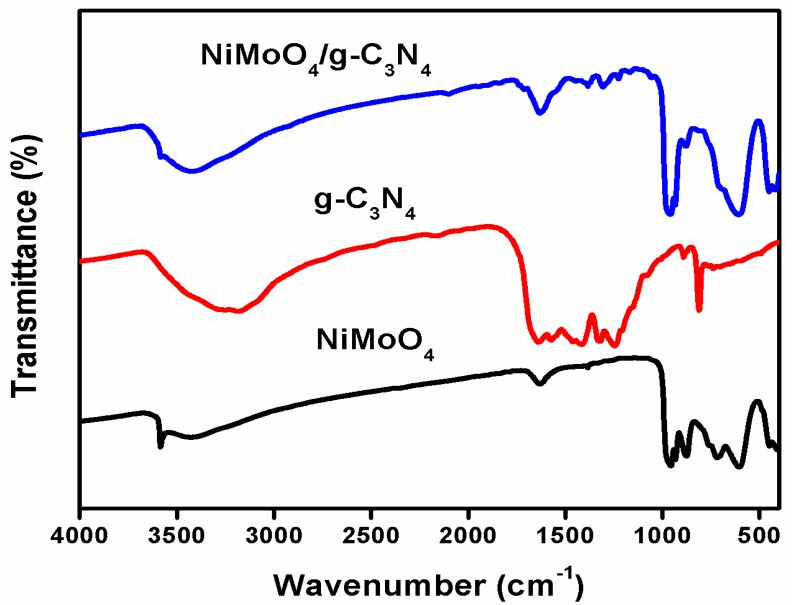
FTIR spectra of NiMoO_4_, g-C_3_N_4_ and NiMoO_4_/g-C_3_N_4_.

FTIR spectra of the as-prepared NiMoO_4_, g-C_3_N_4_, and NiMoO_4_/g-C_3_N_4_ active electrode materials are illustrated in Figure 2. Of interest are the observed vibrational bands at 975 and 862 cm^−1,^ which correspond to the symmetric and antisymmetric stretching vibrations of the Mo-O [31] linkage. The absorbance band at approximately 606 cm^−1^ corresponds to the stretching vibration of Ni-O [32]. Pure g-C_3_N_4_ demonstrates a strong band around 3100–3300 cm^−1^, corresponding to the stretching modes of N–H bonds (–NH_3_ and =NH) of amines [33]. Furthermore, the broad peaks observed in the range of 1200–1680 cm^−1^ are related to the stretching modes of C=N and the heterocyclic aromatic C–N bonds [34]. Moreover, the peak appearing at 811 cm^−1^ is a distinctive breathing mode of triazine units. In the case of NiMoO_4_/g-C_3_N_4_, an increase in the absorbance bands when g-C_3_N_4_ was added to NiMoO_4_ can be observed, indicating the coexistence of the NiMoO_4_ and g-C_3_N_4_ in the composite material, which the XRD data also supports.

### 3.3. Surface Morphology and Elemental Studies

**Figure 3:**
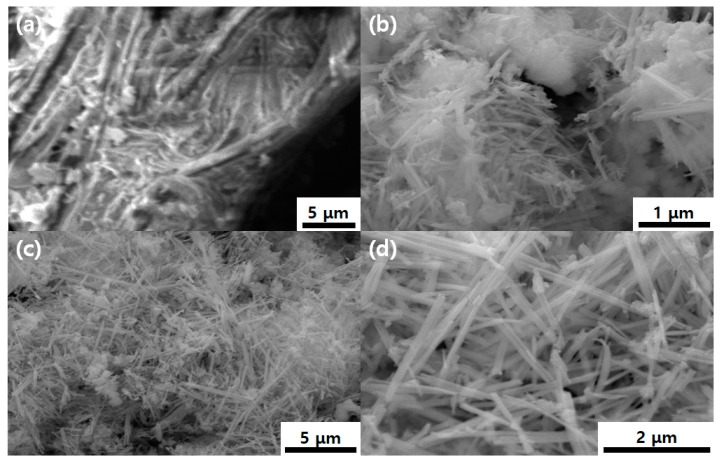
SEM analysis of (**a**) g-C_3_N_4_, (**b**) NiMoO_4_ and (**c**,**d**) NiMoO_4_/g-C_3_N_4_ samples.

**Figure 4:**
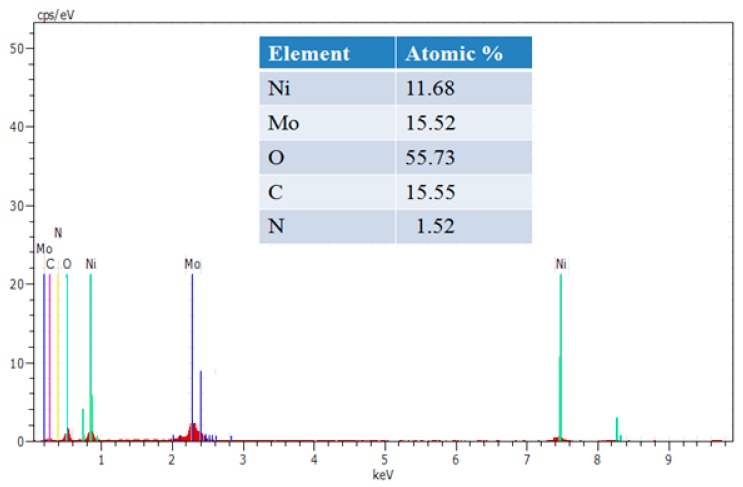
The EDAX spectrum of NiMoO_4_/g-C_3_N_4_ composite.

**Figure 5:**
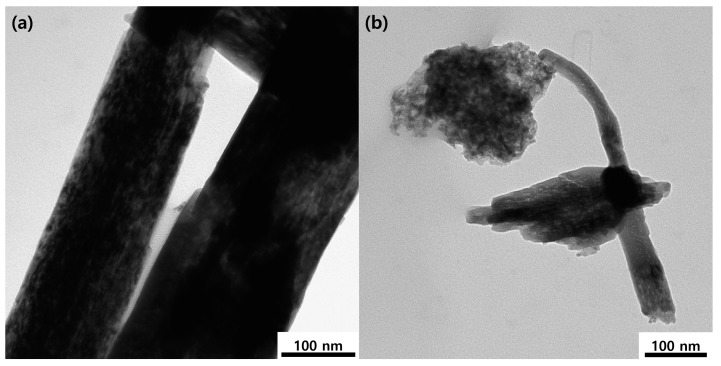
TEM analysis of (**a**,**b**) NiMoO_4_/g-C_3_N_4_ composite.

The morphological characteristics of g-C_3_N_4_, NiMoO_4_, and NiMoO_4_/g-C_3_N_4_ were investigated by scanning electron microscopy (SEM). Characteristic SEM micrographs of g-C_3_N_4_, NiMoO_4_, and NiMoO_4_/g-C_3_N_4_ are displayed in Figure 3_._ The pristine NiMoO_4_ shows a “sponge with fiber”-like morphology, whereas pure g-C_3_N_4_ appears as aggregated particles containing a nanosheet-like morphology. The NiMoO_4_/g-C_3_N_4_ composite exhibits clear nanofiber-like structure (Figure 3c,d). Figure 4 displays the energy dispersive X-ray microanalysis (EDAX) spectrum of NiMoO_4_/g-C_3_N_4_ composite material. The peaks corresponding to Ni, Mo, O, C and N are present with no other peaks observed, indicating the high purity of the as-prepared NiMoO_4_/g-C_3_N_4_ composite. The corresponding atomic (%) ratios of the elements identified by the EDAX spectrum are shown in the inset of Figure 4. The XRD and EDAX results clearly indicate the formation of a NiMoO_4_/g-C_3_N_4_ composite. To further confirm the morphology of the NiMoO_4_/g-C_3_N_4_ composite, transition electron microscopy (TEM) was performed (Figure 5). The TEM images clearly inferred that the morphology of NiMoO_4_/g-C_3_N_4_ composite is of a nanofiber-like structure within a size range of 100 to 200 nm and is attached to the sheet-like structure of g-C_3_N_4_. In comparison with pure NiMoO_4_, the NiMoO_4_/g-C_3_N_4_ composite possesses a clear nanofiber structure, which is favorable for redox reactions. The TEM morphological analyses support the SEM analyses.

### 3.4. CV Studies

**Figure 6:**
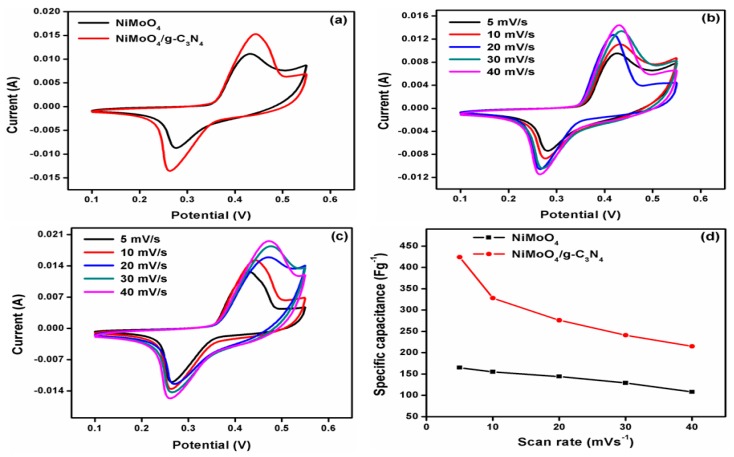
(**a**) Comparison of CV curves of pristine NiMoO_4_ and NiMoO_4_/g-C_3_N_4_ at a scan rate of 10 mVs^−1^, (**b**,**c**) CV curves of pure NiMoO_4_ and composite materials at several scan rates, and (**d**) its corresponding plot of SC with scan rates.

The electrochemical responses of the as-prepared materials were evaluated under CV measurements. Figure 6a shows the CV curves for pristine NiMoO_4_ and NiMoO_4_/g-C_3_N_4_ at a scan rate of 10 mVs^−1^. Both electrode materials exhibit clear redox peaks with the NiMoO_4_/g-C_3_N_4_ electrode material possessing the highest redox peak current value (the peak current values for pure, pristine NiMoO_4_ and NiMoO_4_/g-C_3_N_4_ are 0.011 and 0.015 A at 10 mVs^−1^, respectively), clearly indicating that an improvement in electrochemical performance exists. Figure 6b,c shows the CV curves of pristine NiMoO_4_ and NiMoO_4_/g-C_3_N_4_ at different scan rates of 5 to 40 mVs^−1^. Notably, as the scan rate increases, the anodic and cathodic peak currents increase, and the anodic and cathodic peak potentials shift. This observation is owing to a sluggish ionic diffusion rate preventing electronic neutralization in the Faradaic redox reaction [35]. Figure 6d shows the difference in SC of both the pristine and composite at various scan rates.

### 3.5. GCD Studies

**Figure 7:**
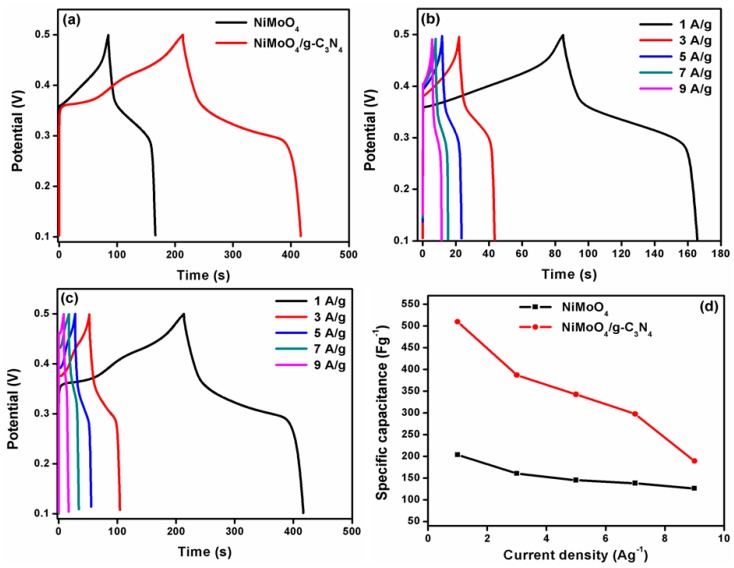
(**a**) Comparative GCD plots of the pristine NiMoO_4_ and NiMoO_4_/g-C_3_N_4_ nanocomposite electrodes at a current of 1 Ag^−1^, (**b**,**c**) GCD curves of the pristine NiMoO_4_ and NiMoO_4_/g-C_3_N_4_ nanocomposite at various current densities, and (**d**) its corresponding plot of SC with related to current densities.

**Figure 8:**
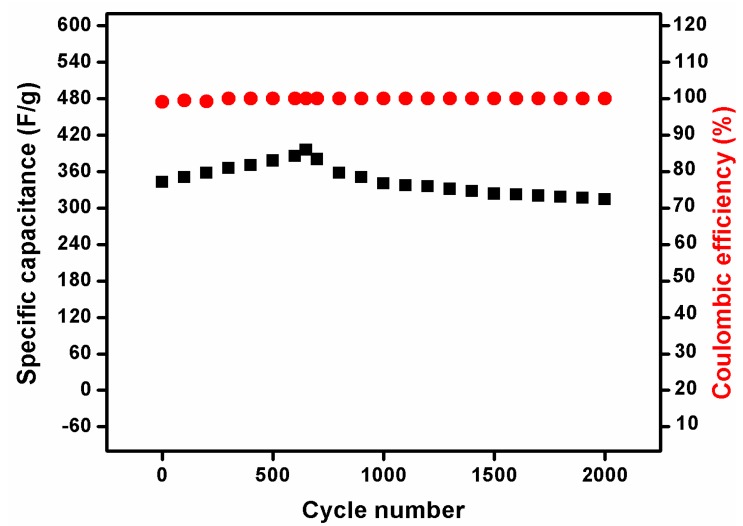
Specific capacitance vs. cycle number and coulombic efficiency vs. cycle number plot of NiMoO_4_/g-C_3_N_4_ nanocomposite electrode.

The GCD performance can be used to identify the stability and time reversibility of the electrode material. The comparison of GCD curves for NiMoO_4_ and NiMoO_4_/g-C_3_N_4_ is displayed in Figure 7a. From the GCD curves, it can be observed that the NiMoO_4_/g-C_3_N_4_ has a higher reversible time than that of NiMoO_4_. The calculated SC values of pristine NiMoO_4_ and NiMoO_4_/g-C_3_N_4_ were found to be 203 and 510 Fg^−1^ at a current of 1 Ag^−1^, respectively. Figure 7b,c illustrates the GCD curves of NiMoO_4_ and NiMoO_4_/g-C_3_N_4_ at several currents from 1 to 9 Ag^−1^. Figure 7d shows the variation of SC with respect to the current. Figure 8 shows the long-term cyclic test of the NiMoO_4_/g-C_3_N_4_ composite electrode material up to 2000 cycles. It can be shown that the NiMoO_4_/g-C_3_N_4_ electrode material retained approximately 91.8% of the SC value after 2000 cycles in comparison with its initial capacitance value. The generated results from the stability test showed that the initial SC value of 342.5 Fg^−1^ gradually increased up to 650 cycles with an SC value of 395 Fg^−1^. In other words, the SC value increased 115% from its initial capacitance value, probably owing to the increased number of active sites available for the electrochemical reactions [36]. The calculated coulombic efficiency was also found to be nearly 100% after 2000 cycles. According to the CV and GCD data, it can be deduced that the NiMoO_4_/g-C_3_N_4_ composite is a suitable electrode material for pseudocapacitor applications.

The energy density (*E*) and power density (*P*) for pseudocapacitors can be calculated from Equations (2) and (3), respectively [37].

*E* = *C*∆*V*^2^ /2 × 3.6 (2)

*P* = *E* × 3600/∆*t*_d_ (3)

where *C* is the SC, ∆*V* is potential used in the GCD measurement, and ∆*t*_d_ is the discharge time (s). The calculated *E* and *P* values of pristine NiMoO_4_ and NiMoO_4_/g-C_3_N_4_ were found to be 4.5 and 11.3 Wh kg^−1^, respectively.

**Figure 9:**
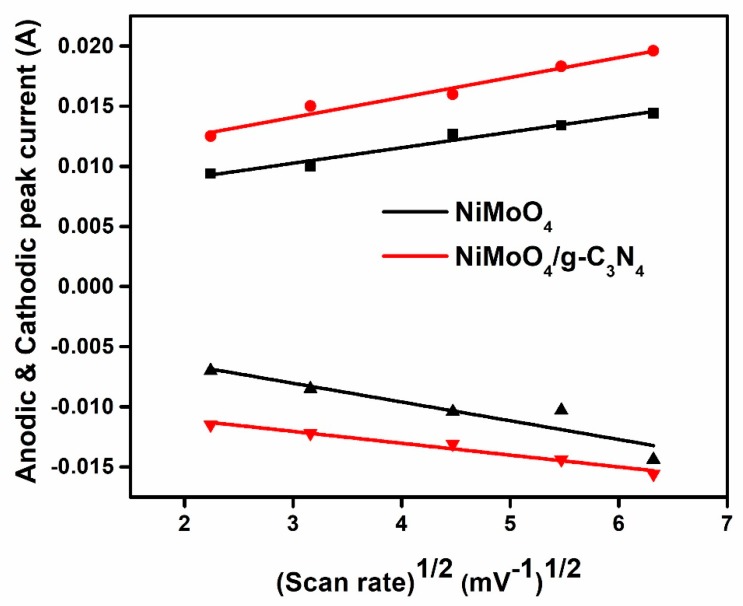
The linear dependence between the cyclic voltammetric anodic and cathodic peak current and the square root of the various scan rates of pristine NiMoO_4_ and NiMoO_4_/g-C_3_N_4_ composite.

Figure 9 shows a linear relationship between the anodic and the cathodic peak currents (*I*_pa.c_), and the square root of the scan rate (values extracted from Figure 6). It is observed that the anodic and cathodic peak currents of NiMoO_4_ and the NiMoO_4_/g-C_3_N_4_ composite follow the Randles–Sevcik equation (Equation (4)) [29],

(4) $$I_{\mathrm{pa},c}=2.687\times 10^{5}\times n^{3/2}\times A\times \surd D\times C\times \surd v$$

where *n* is the number of electrons transferred through reaction, *A* is the working electrode surface area, *D* is the diffusion coefficient, *C* is the electrolyte concentration, and *v* is the scan rate. The Randles–Sevcik equation describes the effect of the scan rate on the anodic or cathodic peak current *I*_pa,c_ in the redox process. Generally, it depends not only on the concentration of the electrolyte but on the diffusional properties of the electrode material and on the scan rate [38,39].

The diffusion coefficient of pristine NiMoO_4_ and NiMoO_4_/g-C_3_N_4_ composite was calculated as 1.539 × 10^−8^ and 3.463 × 10^−8^ cm^2^s^−1^, respectively. The achieved diffusion coefficient values clearly show that the NiMoO_4_/g-C_3_N_4_ composite has a higher value than that of the pristine NiMoO_4_. This is supported by the CV and the GCD results of the NiMoO_4_/g-C_3_N_4_ nanomaterial.

### 3.6. EIS Studies

**Figure 10:**
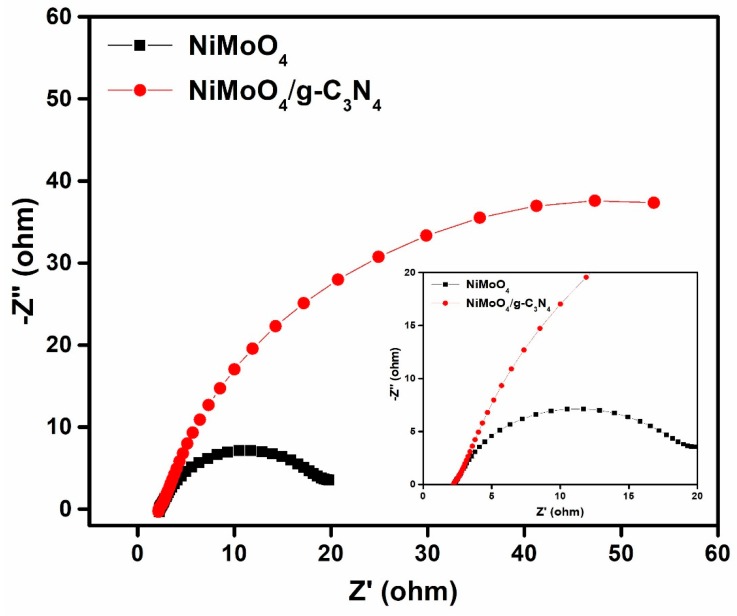
Nyquist plots of pristine NiMoO_4_ and NiMoO_4_/g-C_3_N_4_ electrode materials (inset figure showing the zoomed view of the Nyquist plot).

EIS is an important method to describe the interfacial resistance and the charge-transfer (CT) performance of active materials. Additionally, it can also reveal the pseudocapacitance (*C*_p_), double-layer capacitance (*C*_dl_), bulk resistance (*R*_b_), CT resistance (*R*_ct_) and the series resistance (*R*_s_) that is associated with the summation of the electrode/electrolyte interfacial resistance [40]. The EIS studies for pristine NiMoO_4_ and the NiMoO_4_/g-C_3_N_4_ composite material were carried out in a frequency ranging from 10^5^ Hz to 1 Hz at an amplitude of +0.005 V. The Nyquist plots are illustrated in Figure 10 (the figure inset shows the consequent equivalent circuit model). The impedance spectra in Figure 9 show a semi-circle pattern at higher frequencies and a straight line at lower frequencies. The occurrence of a semi-circle corresponds to the Faradaic redox reaction that occurs on the active material coated area of the pristine NiMoO_4_ and NiMoO_4_/g-C_3_N_4_ composite. The calculated resistance values are shown in Table 1. The calculated *R*_ct_ values for pristine NiMoO_4_ and NiMoO_4_/g-C_3_N_4_ composite were found to be 0.047 and 0.042 Ω, respectively. From Table 1, it is evident that the NiMoO_4_/g-C_3_N_4_ composite exhibited a lower *R*_ct_ value than that of the pristine NiMoO_4_. Furthermore, the EIS results also support CV and GCD results.

## 4. Conclusions

NiMoO_4_/g-C_3_N_4_ composite nanofibers were successfully synthesized using a hydrothermal route. The phase purity and surface morphology were examined by XRD, FTIR, SEM and EDAX analysis. The electrochemical performance of pristine NiMoO_4_ and NiMoO_4_/g-C_3_N_4_ composite materials were evaluated and compared using CV, GCD and EIS studies. The calculated SC value of NiMoO_4_/g-C_3_N_4_ composite was found to be 510 Fg^−1^, whereas pristine NiMoO_4_ had a value of 210 Fg^−1^ at a current 1 Ag^−1^. The NiMoO_4_/g-C_3_N_4_ composite electrode material was subjected to a long-term stability test, and it was found that the as-prepared materials withstood up to 91.8% retention from its initial capacitance value of the charge-discharge curves even after 2000 cycles at a current of 5 Ag^−1^. The NiMoO_4_/g-C_3_N_4_ composite material was found to have a higher energy density value (11.3 Wh kg^−1^) than that of the pristine NiMoO_4_ (4.5 Wh kg^−1^). From the SC results, the NiMoO_4_/g-C_3_N_4_ composite could be potentially used as a supercapacitor electrode material.

## Figures and Tables

**Table 1:** R_s_, R_ct_ and R_b_ values of the pure NiMoO_4_ and NiMoO_4_/g-C_3_N_4_ composite.

Sample	R_s_ (Ω)	R_ct_ (Ω)	R_b_ (Ω)
NiMoO_4_	2.260	0.047	2.307
NiMoO_4_/g-C_3_N_4_	2.212	0.042	2.264

## References

[1] Zhai S., Karahan H.E., Wei L., Qian Q., Harris A.T., Minett A.I., Ramakrishna S., Ng Y.A.K., Chen Y. (2016). Textile Energy Storage: Structural Design Concepts, Material Selection and Future Perspectives. Energy Storage Mater..

[2] Miller J.R., Simon P. (2008). Electrochemical Capacitors for Energy Management. Science.

[3] Simon P., Gogotsi Y. (2008). Materials for Electrochemical Capacitors. Nat. Mater..

[4] Lu X., Yu M., Wang G., Tong Y., Li Y. (2014). Flexible Solid-State Supercapacitors: Design, Fabrication and Applications. Energy Environ. Sci..

[5] Yoo J.J., Balakrishnan K., Huang J., Meunier V., Sumpter B.G., Srivastava A., Conway M., Reddy A.L.M., Yu J., Vajtai R. (2011). Ultrathin Planar Graphene Supercapacitors. Nano Lett..

[6] Huang Y., Liang J., Chen Y. (2012). An Overview of the Applications of Graphene-Based Materials in Supercapacitors. Small.

[7] Zhang L.Y., Tang C.H., Gong H. (2014). Temperature Effect on the Binder-Free Nickel Copper Oxide Nanowires with Superior Supercapacitor Performance. Nanoscale.

[8] Arul N.S., Mangalaraj D., Ramachandran R., Grace A.N., Han J.J.I. (2015). Fabrication of CeO_2_/Fe_2_O_3_ Composite Nanospindles for Enhanced Visible Light Driven Photocatalysts and Supercapacitor Electrodes. J. Mater. Chem. A.

[9] Subramanian V., Hall S.C., Smith P.H., Rambabu B. (2004). Mesoporous Anhydrous RuO_2_ as a Supercapacitor Electrode Material. Solid State Ion..

[10] Ming B., Li J., Kang F., Pang G., Zhang Y., Chen L., Xu J., Wang X. (2012). Microwave–Hydrothermal Synthesis of Birnessite-Type MnO_2_ Nanospheres as Supercapacitor Electrode Materials. J. Power Sources.

[11] Ghosh D., Giri S., Das C.K. (2013). Preparation of CTAB-Assisted Hexagonal Platelet Co(OH)_2_/Graphene Hybrid Composite as Efficient Supercapacitor Electrode Material. Acs Sustain. Chem. Eng..

[12] Yuan C., Zhang X., Su L., Gao B., Shen L. (2009). Facile Synthesis and Self-Assembly of Hierarchical Porous NiO Nano/Micro Spherical Superstructures for High Performance Supercapacitors. J. Mater. Chem..

[13] Xu J., Ding T.T., Wang J., Zhang J., Wang S., Chen C.Q., Fang Y.Y., Wu Z.B., Huo K.F., Dai J.N. (2015). Tungsten Oxide Nanofibers Self-Assembled Mesoscopic Microspheres as High-performance Electrodes for Supercapacitor. Electrochim. Acta.

[14] Chen Y., Meng F., Ma C., Yang Z., Zhu C., Ouyang Q., Gao P., Li J., Sun C. (2012). In-situ diffusion Growth of Fe_2_(MoO_4_)_3_ Nanocrystals on the Surface of α-MoO_3_ Nanorods with Significantly Enhanced Ethanol Sensing Properties. J. Mater. Chem..

[15] Xiao W., Chen J.S., Li C.M., Xu R., Lou X.W. (2010). Synthesis, Characterization, and Lithium Storage Capability of AMoO_4_ (A = Ni, Co) Nanorods. Chem. Materials.

[16] Guo D., Zhang P., Zhang H., Yu X., Zhu J., Li Q., Wang T. (2013). NiMoO_4_ Nanowires Supported on Ni Foam as Novel Advanced Electrodes for Supercapacitors. J. Mater. Chem. A.

[17] Lin L., Liu T., Liu J., Sun R., Hao J., Ji K., Wang Z. (2016). Growth-Controlled Nico_2_s_4_ Nanosheet Arrays with Self-Decorated Nanoneedles for High-Performance Pseudocapacitors. Appl. Surf. Sci..

[18] Senthilkumar B., Sankar K.V., Kalaiselvan R., Danielle M., Manickam M. (2013). Nano α-NiMoO_4_ as a New Electrode for Electrochemical Supercapacitors. RSC Adv..

[19] Raja P., Kannan J., Rahul S., Salunkhe R., Pramanik M., Malgras V., Alshehri S.M., Yamauchi Y. (2015). Synthesis and Characterization of α-NiMoO_4_ Nanorods for Supercapacitor Application. Eur. J. Inorg. Chem..

[20] Wang Z., Wei G., Kun D., Zhao X., Liu M., Wang S., Zhou Y., An C., Zhang J. (2017). Ni Foam-Supported Carbon-Sheathed NiMoO_4_ Nanowires as Integrated Electrode for High-Performance Hybrid Supercapacitors. ACS Sustain. Chem. Eng..

[21] Liu M.C., Kong L.B., Lu C., Ma X.J., Li X.M., Luo Y.C., Kang L. (2013). Design and synthesis of CoMoO_4_–NiMoO_4_·xH_2_O Bundles with Improved Electrochemical Properties for Supercapacitors. J. Mater. Chem. A.

[22] Senthilkumar B., Meyrick D., Leec Y., Kalaiselvan R. (2013). Synthesis and Improved Electrochemical Performances of Nano β-NiMoO_4_–CoMoO_4_·xH_2_O Composites for Asymmetric Supercapacitors. RSC Adv..

[23] Ren W., Guo D., Zhuo M., Guan B., Zhang D., Li Q. (2015). NiMoO_4_@Co(OH)_2_ Core/Shell Structure Nanowire Arrays Supported on Ni Foam for High-Performance Supercapacitors. RSC Adv..

[24] Theerthagiri J., Senthil R.A., Priya A., Madhavan J., Ashokkumar M. (2015). Synthesis of a Visible-Light Active V_2_O_5_-g-C_3_N_4_ Heterojunction as an Efficient Photocatalytic and Photoelectrochemical Material. New J. Chem..

[25] Zheng Y., Jiao Y., Chen J., Liu J., Liang J., Du A., Zhang W., Zhu Z., Smith S.C., Jaroniec M. (2011). Nanoporous Graphitic-C_3_N_4_@Carbon Metal-Free Electrocatalysts for Highly Efficient Oxygen Reduction. J. Am. Chem. Soc..

[26] Zheng Y., Liu J., Liang J., Jaroniec M., Qiao S.Z. (2012). Graphitic Carbon Nitride Materials: Controllable Synthesis and Applications in Fuel Cells and Photocatalysis. Energy Environ. Sci..

[27] Zhang J., Ding J., Li C., Li B., Li D., Liu Z., Cai Q., Zhang J., Liu Y. (2017). Fabrication of Novel Ternary Three-Dimensional RuO_2_/Graphitic-C_3_N_4_@Reduced Graphene Oxide Aerogel Composites for Supercapacitors. ACS Sust. Chem. Eng..

[28] Zhang N., Chen C., Chen Y., Chen G., Liao C., Liang B., Zhang J., Li A., Yang B., Zheng Z. (2018). Ni_2_P_2_O_7_ Nanoarrays with Decorated C_3_N_4_ Nanosheets as Efficient Electrode for Supercapacitors. ACS Appl. Energy Mater..

[29] Theerthagiri J., Thiagarajan K., Senthilkumar B., Khan Z., Senthil R.A., Arunachalam P., Madhavan J., Ashokkumar M. (2017). Synthesis of Hierarchical Cobalt Phosphate Nanoflakes and their Enhanced Electrochemical Performances for Supercapacitor Applications. ChemistrySelect.

[30] Tian Y., Chang B., Fu J., Zhou B., Liu j., Xi F., Dong X. (2014). Graphitic Carbon Nitride/Cu_2_O Heterojunctions: Preparation, Characterization, and Enhanced Photocatalytic Activity Under Visible Light. J. Solid State Chem..

[31] Klissurski D., Mancheva M., Iordanova R., Tyuliev G., Kunev B. (2006). Mechanochemical Synthesis of Nanocrystalline Nickel Molybdates. J. Alloy Compd..

[32] Wan H.Z., Jiang J.J., Ji X., Miao L., Zhang L., Xu K., Chen H.C., Ruan Y.J. (2013). Rapid Microwave-Assisted Synthesis NiMoO_4_·H_2_O Nanoclusters for Supercapacitors. Mater. Lett..

[33] Yang F., Kuznietsov V., Lublow M., Merschjann C., Steigert A., Klaer J., Thomas A., Niedrig T.S. (2012). Solar Hydrogen Evolution Using Metal-Free Photocatalytic Polymeric Carbon Nitride/CuInS_2_ Composites as Photocathodes. J. Mater. Chem. A.

[34] He Y., Zhang L., Wang X., Wu Y., Lin H., Zhao L., Weng W., Wan H., Fan M. (2014). Enhanced Photodegradation Activity of Methyl Orange Over Z-scheme Type MoO_3_-g-C_3_N_4_ Composite Under Visible Light Irradiation. RSC Adv..

[35] Li X., Xiong S., Li J., Bai J., Qian Y. (2012). Mesoporous NiO Ultrathin Nanowire Networks Topotactically Transformed From α-Ni(OH)_2_ Hierarchical Microspheres and their Superior Electrochemical Capacitance Properties and Excellent Capability for Water Treatment. J. Mater. Chem..

[36] Bi Y., Nautiyal A., Zhang H., Luo J., Zhang X. (2018). One-Pot Microwave Synthesis of NiO/MnO_2_ Composite as a High-Performance Electrode Material for Supercapacitors. Electrochim. Acta.

[37] Thiagarajan K., Theerthagiri J., Senthil R.A., Arunachalam P., Madhavan J., Ghanem M.A. (2018). Synthesis of Ni_3_V_2_O_8_@Graphene Oxide Nanocomposite as an Efficient Electrode Material for Supercapacitor Applications. J. Solid State Electrochem..

[38] Zanello P. (2003). Inorganic Electrochemistry: Theory, Practice and Application.

[39] Thiagarajan K., Theerthagiri J., Senthil R.A., Madhavan J. (2017). Simple and Low Cost Electrode Material Based on Ca_2_V_2_O_7_/PANI Nanoplatelets for Supercapacitor Applications. J. Mater. Sci. Mater. Electron..

[40] Liu C.G., Liu M., Li F., Cheng H.M. (2008). Frequency Response Characteristic of Single-Walled Carbon Nanotubes as Supercapacitor Electrode Material. Appl. Phys. Lett..

